# Supplementary material for: RNA editing of AZIN1 coding sites is catalyzed by ADAR1 p150 after splicing
Source: J Biol Chem. 2023 May 18;299(7):104840. doi: 10.1016/j.jbc.2023.104840 (PMC10404624; doi:10.1016/j.jbc.2023.104840)
Supplement: Supporting Figures S1–S3 [file mmc1.pdf]

SUPPORTING INFORMATION

RNA editing of AZIN1 coding sites is catalyzed by ADAR1 p150 after splicing

Yanfang Xing[1,†], Taisuke Nakahama[1,2,3,†,*], Yuke Wu[1,4], Maal Inoue[1], Jung In Kim[1], Hiroyuki Todo[1], Toshiharu Shibuya[1], Yuki Kato[1,2], Yukio Kawahara[1,2,3,4,5,*]

[1]Department of RNA Biology and Neuroscience, Graduate School of Medicine, Osaka University, Suita, Osaka 565-0871, Japan

[2]Integrated Frontier Research for Medical Science Division and RNA Frontier Science Division, Institute for Open and Transdisciplinary Research Initiatives (OTRI), Osaka University, Suita, Osaka 565-0871, Japan

[3]Center for Infectious Disease Education and Research (CiDER), Osaka University, Suita, Osaka 565-0871, Japan

[4]Graduate School of Frontier Biosciences, Osaka University, Suita, Osaka 565-0871, Japan

[5]Genome Editing Research and Development Center, Graduate School of Medicine, Osaka University, Suita, Osaka 565-0871, Japan

*Corresponding authors: Taisuke Nakahama and Yukio Kawahara

Email: nakahama@rna.med.osaka-u.ac.jp and ykawahara@rna.med.osaka-u.ac.jp

[†]These authors equally contributed to this work.

This file includes:

Supplementary Figures S1 to S3

Supplementary Figures

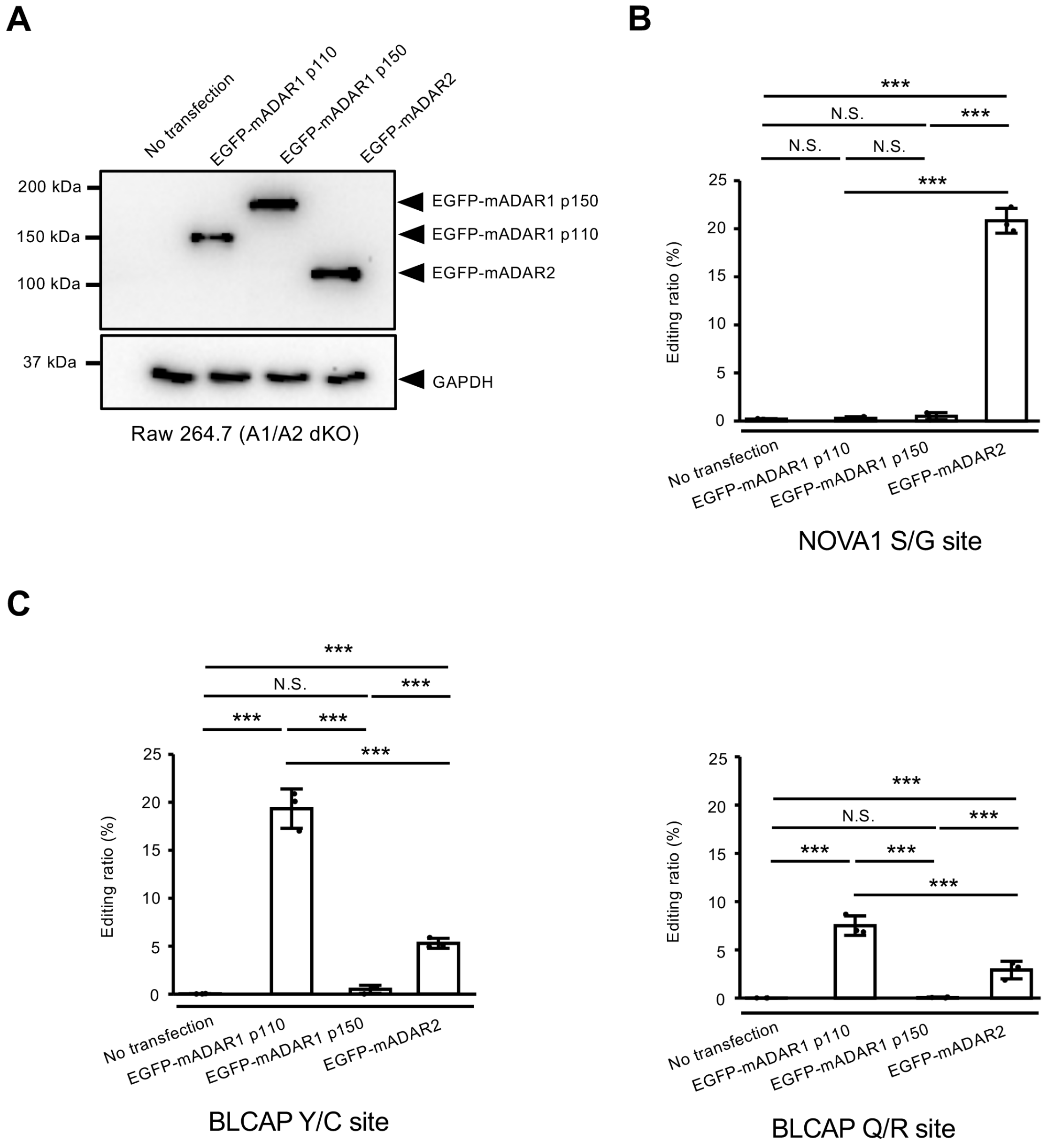

Supplementary Figure S1. ADAR isoform–specific RNA editing. (**A**) The expression of EGFP-tagged mouse ADAR1 p110 (mADAR1 p110), mADAR1 p150, and mADAR2 proteins in *Adar1*/*Adar2* double-knockout (A1/A2 dKO) Raw 264.7 cells was detected using anti-GFP antibody. The expression of GAPDH protein is shown as a reference. (**B, C**) Editing ratios at NOVA1 S/G (**B**), BLCAP Y/C, and BLCAP Q/R (**C**) sites were compared among indicated EGFP-tagged ADAR isoforms expressed in A1/A2 double knockout (dKO) Raw 264.7 cells. Values represent the mean ± SD (n = 3 for each group; Tukey's honest significant difference test, ****p* < 0.005, N.S., not significant).

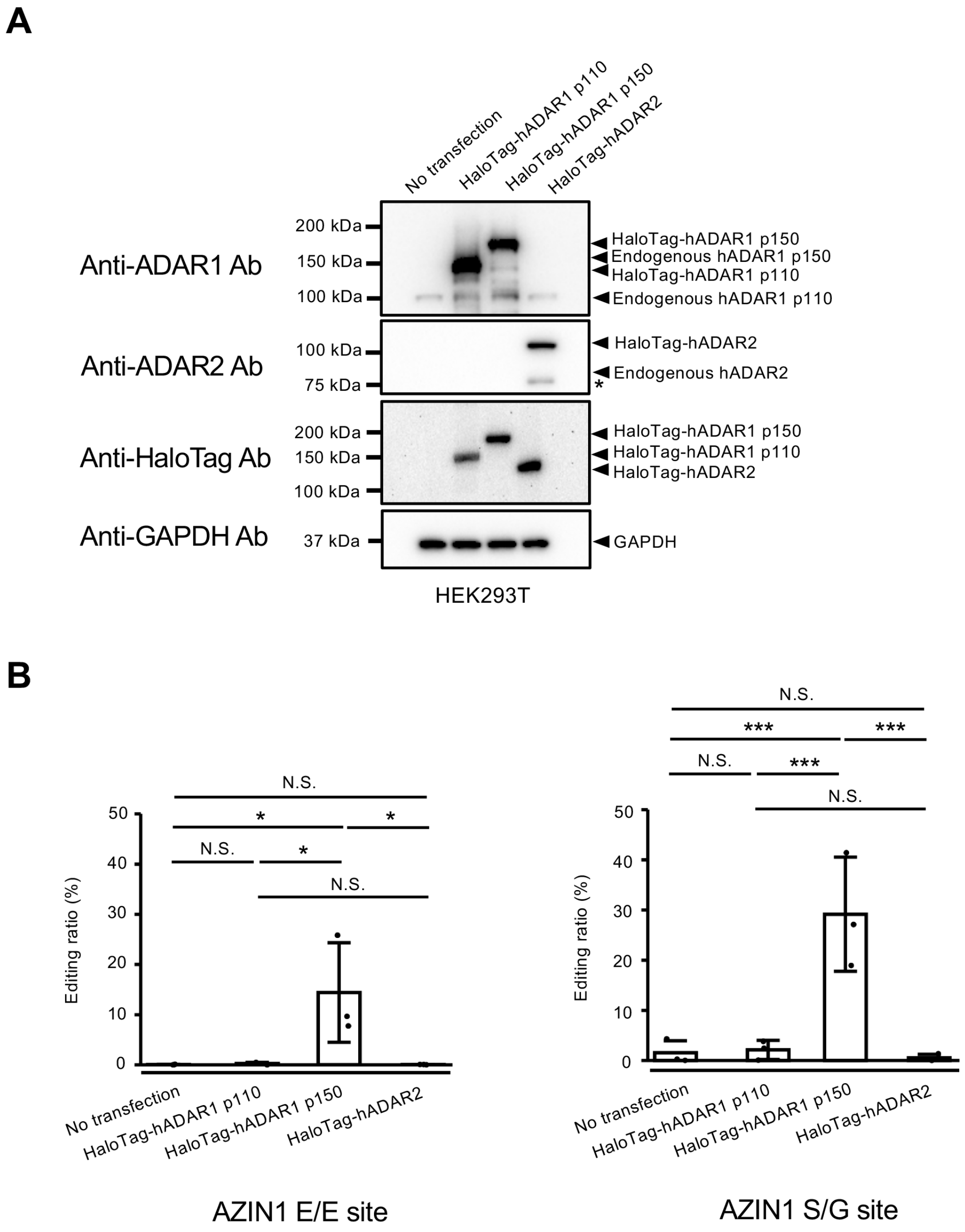

Supplementary Figure S2. ADAR1 p150–specific RNA editing of human AZIN1 mRNA. (**A**) The expression of HaloTag-fused human ADAR (hADAR) proteins expressed in human HEK293T cells was detected using anti-ADAR1, anti-ADAR2, or anti-HaloTag antibodies. The expression of GAPDH protein is shown as a reference. The truncated ADAR2 expression is indicated with an asterisk (*). (**B**) Editing ratios at AZIN1 E/E and S/G sites were compared among indicated HaloTag-fused hADAR isoforms expressed in HEK293T cells. Values represent the mean ± SD (n = 3 for each group; Tukey's honest significant difference test, *$p < 0.05$, ***$p < 0.005$, N.S., not significant).

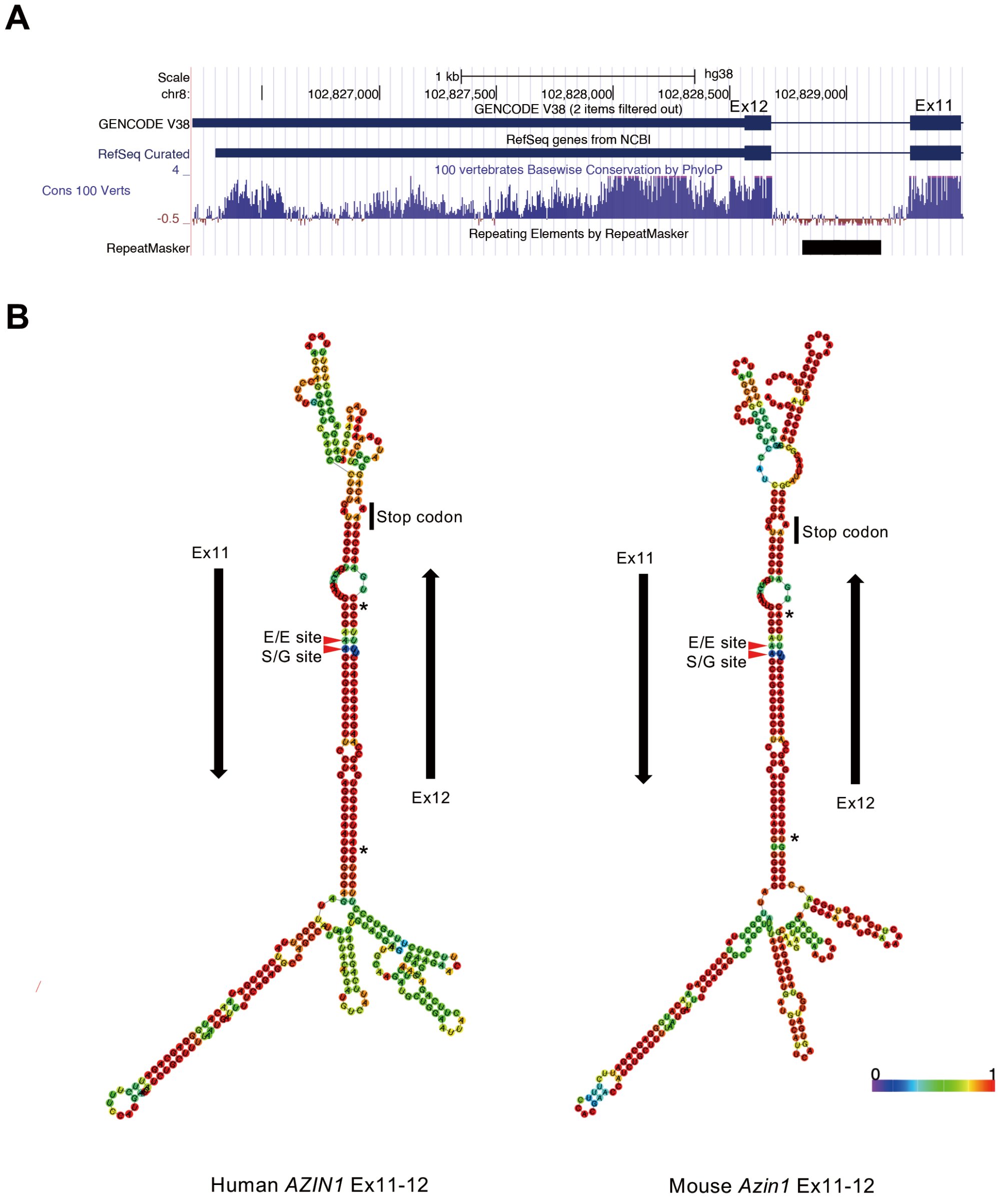

Supplementary Figure S3. Conservation of the secondary structure required for AZIN1 RNA editing between human and mouse. (**A**) The genomic region encompassing exon 11 (Ex11), intron 11, and exon 12 (Ex12) of human AZIN1 was analyzed using a University of California Santa Cruz (UCSC) genome browser. Conservation among 100 vertebrates is shown. (**B**) The secondary structure formed between Ex11 and partial Ex12 of human AZIN1 (left panel) or mouse Azin1 (right panel) mRNAs was estimated using a RNAfold web server and colored by base-pairing probabilities. The two editing sites are indicated by red arrowheads. Nucleotides that differ between human and mouse are indicated with an asterisk (*).
